# The efficacy of Cognitive training in patients with VAsCular Cognitive Impairment, No dEmentia (the Cog-VACCINE study): study protocol for a randomized controlled trial

**DOI:** 10.1186/s13063-016-1523-x

**Published:** 2016-08-05

**Authors:** Yi Tang, Zude Zhu, Qing Liu, Fang Li, Jianwei Yang, Fangyu Li, Yi Xing, Jianping Jia

**Affiliations:** 1 Department of Neurology, Xuan Wu Hospital, Capital Medical University, 45 Changchun Street, Beijing, 100053 China; 2 Collaborative Innovation Center for Language Competence, Jiangsu Normal University, Xuzhou, Jiangsu China; 3 Department of Geriatric Medicine, Fu Xing Hospital, Capital Medical University, Beijing, China; 4 Department of Neurology, Beijing Friendship Hospital, Capital Medical University, Beijing, China

**Keywords:** Cognitive training, Magnetic resonance imaging, Neuroplasticity, No dementia, Protocol, Randomized controlled clinical trial, Vascular cognitive impairment

## Abstract

**Background:**

Vascular cognitive impairment, no dementia (VCIND) refers to cognitive deficits associated with underlying vascular causes that fall short of a dementia diagnosis. There is currently no treatment for VCIND. Computerized cognitive training, which has significantly improved cognitive function in healthy older adults and patients with cognitive impairment has not yet been applied to VCIND.

**Methods/Design:**

The proposed study is a three-center, double-blinded, randomized controlled trial that will include 60 patients with VCIND. The patients will be randomized to either a training or a control group. The intervention is internet-based cognitive training performed for 30 min over 35 sessions. Neuropsychological assessment and functional and structural MRI will be performed before and after 7 weeks training. Primary outcomes are global cognitive function and executive function. Secondary outcome measures are neuroplasticity changes measured by functional and structural MRI.

**Discussion:**

Applying an internet-based, multi-domain, adaptive program, this study aims to assess whether cognitive training improves cognitive abilities and neural plasticity in patients with subcortical VCIND. In addition to the comprehensive assessment of the participants by neuropsychological tests, cerebrovascular risk factors and apolipoprotein E genotyping, neuroplasticity will be used as an evaluation outcome in this study for, to our knowledge, the first time. The combination of functional and structural MRI and neuropsychological tests will have strong sensitivity in evaluating the effects of cognitive training and will also reveal the underlying mechanisms at work.

**Trial registration:**

ClinicalTrials.gov NCT02640716. Retrospectively registered on 21 December 2015.

## Background

Cerebrovascular disease remains the second leading contributor to disability in older people in low- and middle-income countries [1]. In addition to causing physical disabilities, cerebrovascular disease has long been recognized as an important cause of cognitive impairment. The term ‘vascular cognitive impairment’ was introduced to explain all forms of cognitive dysfunction caused by cerebrovascular disease, ranging from mild cognitive dysfunction (vascular cognitive impairment, no dementia, VCIND) to overt dementia (vascular dementia) [2].

Vascular cognitive impairment, no dementia, which is also termed mild vascular cognitive disorder [3], refers to cognitive deficits associated with underlying vascular causes that fall short of a diagnosis of dementia [4, 5]. According to the China Cognition and Aging Study, VCIND is the most common subtype of mild cognitive impairment in China, accounting for 42.0 % of the total cases [6]. Fifty percent of patients with VCIND in the Canadian Study of Health and Aging progressed to dementia over 5 years of follow-up [7].

Although early intervention of VCIND holds the potential to delay or even reverse cognitive impairment, no treatment is available to prevent further decline in patients with VCIND [8]. Because of the significant heterogeneity of VCIND, clinical intervention trials need to focus on a particular subtype to obtain an accurate efficacy evaluation [8]. Owing to its relatively homogeneous features, VCIND caused by subcortical ischemic small vessel disease (subcortical VCIND) is a suitable category for intervention trials.

Executive dysfunction is the characteristic impairment in subcortical vascular cognitive impairment [9–12]. This is mainly due to disruption of the frontal-subcortical circuits, which are particularly vulnerable to ischemic lesions. The occurrence of executive dysfunction also provides a clue for cognitive training. Cognitive training refers to an intervention that provides structured practice on tasks relevant to different aspects of cognitive function, such as memory and executive function. Training-related cognitive improvements in healthy older people have been found [13], with the effect being preserved 6 months after training [14].

Similar training-related cognitive improvements have also been observed in patients with cognitive impairment [15–18] with a trend to apply multi-domain programs [19]. Those enhancements were preserved 6 months [20] and 28 months [21] after training. Although inconsistencies have been found in behavioral or clinical symptom evaluation [16], recent studies using functional or structural MRI have more consistently shown neural plasticity in patients with amnestic mild cognitive impairment [22, 23]. To date, no cognitive intervention study on VCIND has been published. Whether and how cognitive training improves cognitive function in patients with VCIND remains largely unknown.

Brain structural and functional plasticity are the underlying mechanism of cognitive training [24]. Structural plasticity included increased cortical thickness [25] or improved white matter integrity [26]. Functional plasticity included changes in brain function [14] or cerebral blood flow [27]. This training-related neuroplasticity has also been observed in patients with cognitive impairment [22]. Therefore, in addition to unveiling the mechanism, neural plasticity change could also be used to evaluate the efficacy of cognitive training.

This trial is the first study to test the efficacy of cognitive training on VCIND using a double-blinded, randomized controlled trial design. To evaluate the efficacy of cognitive training, both traditional outcomes, such as neuropsychological assessment, and neuroplasticity outcomes, such as brain microstructure index, will be used. Neuroplasticity outcomes might identify training-related changes with more sensitivity. In addition, this study seeks to investigate individual differences in training outcome and its relationship with apolipoprotein E genotyping and cerebrovascular risk factors.

## Methods/Design

### Study design

This study will be implemented as a three-center double-blinded randomized trial. The study was registered under clinicaltrials.gov (NCT02640716). This study will be reported in accordance with both the CONSORT statement and the CONSORT statement for non-pharmacological interventions [28, 29].

The primary objective is to assess whether cognitive training in patients with subcortical VCIND improves their cognitive abilities. The second objective is to evaluate the effect of cognitive training on neural plasticity. Finally, possible genetic and plasma biomarkers related to the effect of the training will be examined.

### Participants

Sixty patients with subcortical VCIND will be recruited on fulfillment of the inclusion criteria. The patients will be randomly allocated into the experimental group or the control group. Patients will be recruited in neurology clinics at Beijing Friendship Hospital, Xuan Wu Hospital, and the geriatric clinic at Fu Xing Hospital, Capital Medical University.

### Inclusion criteria

- Literate Han Chinese, aged 50 years or older, with a consistent caregiver who accompanies the subject at least 4 days per week
- Patient or informant report of cognitive impairment involving memory or other cognitive domains lasting for at least 3 months
- Neither normal nor demented according to the criteria of the Diagnostic and Statistical Manual of Mental Disorders, Fourth Edition [30, 31], with a Clinical Dementia Rating ≥0.5 on at least one domain [32] and a global score ≤0.5; a Mini-Mental State Examination score ≥20 (primary school), or ≥24 (junior school or above) [33, 34]
- Normal or slightly impaired activities of daily living as defined by a total score of ≤1.5 on the three functional Clinical Dementia Rating domains (home and hobbies, community affairs, and personal care) [34]
- MRI entry criteria:
  - o At least three supratentorial subcortical small infarcts (3–20 mm in diameter), with or without white matter lesions of any degree; or moderate to severe white matter lesions (score ≥2 according to the Fazekas rating scale) [35] with or without small infarcts
  - o Absence of cortical and watershed infarcts, hemorrhages, hydrocephalus, and white matter lesions with specific causes (e.g., multiple sclerosis)
  - o No hippocampal or entorhinal cortex atrophy (score 0 according to the medial temporal lobe atrophy scale of Scheltens) [36]

### Exclusion criteria

- Severe aphasia, physical disabilities, or any other factor that might preclude completion of neuropsychological testing
- Clinically significant gastrointestinal, renal, hepatic, respiratory, infectious, endocrine, or cardiovascular system disease; cancer; alcoholism; drug addiction
- Disorders other than subcortical VCIND that might affect cognition; a Hamilton Depression Scale score >17 or schizophrenia; new strokes within 3 months before baseline; inherited or inflammatory small vessel disease
- Use of medications that may affect cognitive functioning, including tranquilizers, anti-anxiolytics, hypnotics, nootropics, and cholinomimetic agents; and inability to undergo brain MRI

### Randomization

Participants will be randomly allocated to either the intervention group or the control group in a ratio of 1:1. After participants have given their informed consent, randomization will be performed by an independent statistician who is blinded to the patient interventions using simple randomization of random number table method in SAS software (SAS Institute, Inc., Cary, NC, USA). Afterwards, the sealed randomization codes and intervention number are sent out to each center. Blinding will be broken only if a participant needs emergency treatment. Once the blinding is broken, the participant will be managed as off-trial.

### Blinding

Patients, caregivers, radiologists, statisticians, and neuropsychologists who measure the outcomes will be blinded to the randomization status. Blinding will also be maintained for data management, outcome assessment, and data analysis.

### Intervention

Previous studies showed that training approaches with multi-domain, personalized, and adaptive training features are more effective [19, 37, 38]. Therefore, cognitive training will be a computerized multi-domain adaptive training program in this trial. Training paradigms that were successfully used in previous studies will be adopted [19, 37], including processing speed, attention, long-term memory, working memory, flexibility, calculation, and problem solving. Specific training paradigms include a time perception task, visual search task, rapid serial presentation task, delayed match to sample task, paired-associate recall task, attention span task, digit span task, go–no go task, Stroop task, task switching, and *n*-back working memory task. To enable adaptive training, each task was designed with several difficulty levels. Based on previous tests with a large size sample, the tasks will be further grouped in each domain with varied task difficulty. At the beginning, assignment tasks from these domains will be similar across participants. On each training day, five tasks (2 min per task, each three times, in total 30 min per day) will be assigned. Within each task, high accuracy (>80 %) is required to upgrade. To manipulate the adaptive change, the number of types of stimuli, the presentation probability of each type of stimuli, and the size and duration of a stimulus were systematically set. To keep a systematical setting, only one parameter will be changed, while the other parameters will be kept as constant in one level upgraded. Once the task performance is higher than 80 % of the norm performance of a normal aging population, the task will be replaced by a harder task from the same domain. The training is thus also adaptive at participant level, with a similar setup but personalized progress across participants.

For the control group, processing speed and attention tasks are included, with five tasks and 30 min training in each day. Importantly, a fixed, primary difficulty level for all participants in the control group is set. The training will be completed at home and supervised by an independent neurologist over the internet (www.66nao.com).

### Primary outcome measures

The primary outcome measures are global cognitive function measured by the Montreal Cognitive Assessment and executive function measured by the Trail Making Test B-A.

### Secondary outcome measures

The secondary outcome measure is neuroplasticity changes, as measured by MRI. Specifically, the brain functional response, including regional activation magnitude and functional connectivity across regions will be assessed. For fMRI, both before and after the trial, a resting state scan, a scan with a cognitive control task, and a scan with an episodic memory task will be included. The brain response change during the tasks and functional connectivity across regions in the task and the resting state sessions will be examined between groups. The structural change, including gray matter volume, measured by voxel-based morphometrics, and white matter microstructure, measured by diffusion tensor imaging, will be assessed.

### Data collection

At screening, the following data will be collected: demographic data (sex, age, education, and occupation); medical history; concomitant medications; findings from a complete physical examination and neurological examination; neuropsychological assessment; blood analysis; inclusion and exclusion criteria will be assessed; functional and structural brain MRI will be carried out if the patient meets the inclusion and exclusion criteria.

Blood samples will be collected and the following tests will be carried out: complete blood count, liver enzymes, kidney function, blood glucose, cholesterol, homocysteine, and apolipoprotein E genotyping.

**Fig. 1:**
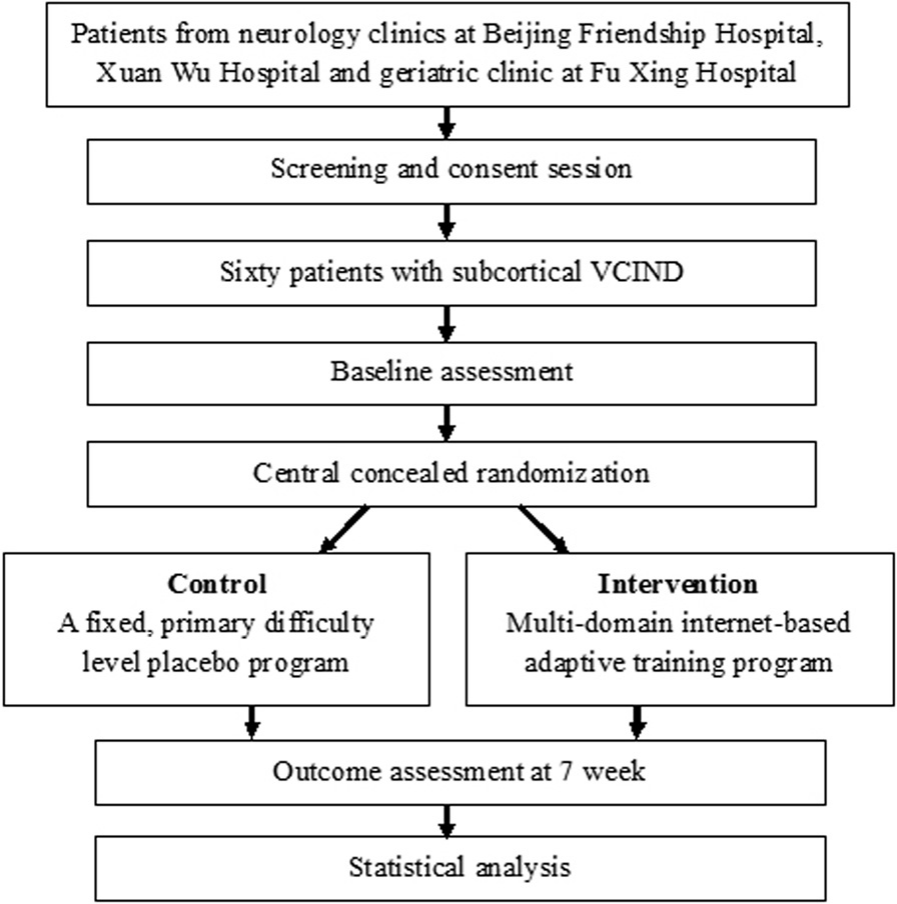
Overview of the flow of participants through the trial

A follow-up assessment will be scheduled after 7 weeks of cognitive training. Data from the following examinations will be collected: physical and neurological examinations; neuropsychological assessment; functional and structural brain MRI (Fig. 1).

### Neuropsychological assessment

The neuropsychological evaluation includes the administration of several commonly used measures (tests, interview, and questionnaires) of cognitive and daily functions. Measures include the Mini-Mental State Examination, the Montreal Cognitive Assessment, the Clinical Dementia Rating scales, Digit Span, Trail Making Test A and B, the WHO-UCLA Auditory Verbal Learning Test, the Boston Naming Test, the Hachinski Ischemic Scale, the Geriatric Depression Scale, the Neuropsychiatric Inventory, and an activities of daily living assessment. Furthermore, in the executive domain, a task-switching paradigm that includes non-switch and switch blocks will be administered inside the scanner. In the memory domain, an episodic memory task that includes a learning session and a test session will be administered both outside and inside the scanner.

### MRI protocol

Patients will undergo brain MRI at baseline and 7 weeks after training. The brain MRI will be performed using an optimized protocol, with the same 3 T MRI scanner (Magnetom Skyra, Siemens Healthcare, Erlangen, Germany). Following a pilot scan, a three-dimensional magnetization-prepared rapid gradient-echo scan will be performed (repetition time = 1690 ms, echo time = 2.56 ms, flip angle = 12°, 1 mm isotropic voxels covering the whole brain). T2*-weighted functional images will be collected using a gradient-echo echo-planar imaging sequence (33 interleaved slices, repetition time = 2000 ms, echo time = 30 ms, flip angle = 83°, field of view = 224 mm^2^, matrix = 64 × 64, 3.5 mm isotropic voxels). Diffusion tensor imaging will use a double spin-echo echo-planar imaging sequence (repetition time = 8000 ms, echo time = 96 ms, flip angle = 90°, field of view = 224 mm^2^, in-plane resolution =1.75 × 1.75 mm voxels, 54 contiguous 2 mm thick axial slices). Diffusion tensor images will be acquired with 64 noncollinear encoding directions (*b* = 1000 s/mm^2^) and 11 images without diffusion weighting (*b* = 0 s/mm^2^, *b*_0_). All scans will be reviewed qualitatively by a radiologist to screen for possible brain lesions or structural abnormalities. Diffusion tensor imaging and functional MRI data collected during the episodic memory task will be analyzed using the FSL package (FMRIB Analysis Group, Oxford, UK). Brain activation and connectivity changes before and after training will be compared between experimental and control groups. By using tract-based spatial statistics implanted in FSL, fractional anisotropy and mean diffusivity values in central white matter tracts will be measured and compared before and after training.

### Apolipoprotein E genotyping

High-molecular-weight DNA will be isolated from peripheral blood leukocytes using a QIAamp DNA Blood Kit (QIAGEN, Valencia, CA, USA). Genomic DNA will be amplified by PCR using the primers ApoE-5′: 5′-AGACGCGGGCACGGCTGTCCAAGGA-3′ and ApoE-3′: 5′-CCCTCGCGGGCCCCGGCCTGGTACAC-3′ with the following conditions: denaturation at 95 °C for 5 minutes, followed by 30 cycles at 95 °C for 30 s, annealing at 60 °C for 30 s, and extending at 72 °C for 30 s. The PCR products will be digested with *Hha*I (R0139S, New England BioLabs, Beverly, MA, USA). The fragments will be separated by electrophoresis on a 20 % polyacrylamide gel and visualized using GelGreen™ nucleic acid gel stain (89139–144, Biotium, Hayward, CA, USA).

### Data monitoring

All participants from three centers will be evaluated by the same trained neuropsychologists and undergo brain MRI using the same MRI scanner. Imaging data are checked for quality and protocol conformity after each scanning session.

### Sample size estimates

In preliminary tests, the observed mean difference in change from baseline in the DST, WHO-UCLA Auditory Verbal Learning Test, and Boston Naming Test scores between training and control groups were 14.92, 3.35, and 7.59, respectively, and the standard deviations were 18.41, 4.05, and 9.06, respectively. Based on these data, sample sizes of 48, 46, and 46, respectively, were needed to obtain a statistical power of 80 % with a significance level of 5 %. We used the largest sample size of 48, and the inclusion number has been set to 60 patients, allowing for a maximum dropout rate of 20 %. Power calculations are based on the primary outcome measures, which are assessed by neuropsychological tests. The statistical power measures will be calculated using the POWER procedure in SAS® Version 9.3.

### Statistical analysis

Performance changes in the assessment scores related to global cognitive function (Montreal Cognitive Assessment) and executive function (Trail Making Test B-A) are the primary outcome measures of interest. An independent-sample *t* test will be used separately for the assessment criterion and the rating scales of function, to ensure that baseline levels are comparable between the training and control groups. A paired-samples *t* test will be conducted to compare the changes in scores in the trained tasks to investigate the training efficacy. The performance change of the trained tasks will be further correlated with the neuropsychological change. To test whether the training will improve neuropsychological performance in the control group, correlation analysis will be conducted between the performance of trained tasks and the neuropsychological change. To determine the training transfer effect, a series of 2 × 2 ANOVAs will be conducted with group and time points as the two factors. For all analyses, significance levels will be set to 0.05, and effect sizes refer to partial *η*-square values. Statistical analysis will be conducted using SPSS20.0 software. Imaging data will be analyzed using FSL to detect any changes in brain function and structure due to cognitive training.

## Discussion

To our knowledge, this trial is the first to evaluate the cognitive training efficacy in VCIND patients. This study has several strengths. First, the multi-domain cognitive training applied in this study is highly adaptive and internet-based. Such training is believed to be effective in enhancing cognitive function. Conducting training on the internet is convenient, allowing patients to train at home and doctors to easily manage the training protocol and monitor training progress.

Second, functional and structural plasticity are used as the evaluation outcome measures. Compared with the traditional efficacy evaluations, which use neuropsychological tests, the combination of neuroplasticity and neuropsychological tests in this study will be more sensitive in testing cognitive training efficacy, because smaller training effects could be detected by MRI indices, such as brain activity and connectivity [16]. In this trial, by including the functional and structural MRI measurements, a further purpose is to reveal the underlying mechanism at work. For instance, fMRI measurement would provide evidence to distinguish whether neural efficiency was improved or the brain function was reorganized to achieve cognitive enhancement. The structural MRI measurement would provide evidence on the structural constraint on functional change due to cognitive training.

Another strength of this trial is the comprehensive assessment of the participants on multiple levels, including neuropsychological tests, neuroplasticity analysis, cerebrovascular risk factors, and apolipoprotein E genotyping. This comprehensive assessment will be able to identify possible biomarkers involved in the effects of cognitive training in VCIND.

## Trial status

This trial is currently recruiting participants.

## Abbreviations

Cog-VACCINE, Cognitive training in patients with VAsCular Cognitive Impairment, No dEmentia; CONSORT, Consolidated Standards of Reporting Trials; MRI, magnetic resonance imaging; PCR, polymerase chain reaction; VCIND, vascular cognitive impairment, no dementia
